# The effect of nilotinib plus arsenic trioxide on the proliferation and differentiation of primary leukemic cells from patients with chronic myoloid leukemia in blast crisis

**DOI:** 10.1186/s12935-015-0158-4

**Published:** 2015-02-04

**Authors:** Wei Wang, Fei-fei Lv, Yan Du, Nannan Li, YaLing Chen, LiHong Chen

**Affiliations:** Department of Hematology, Southeast Hospital Affiliated to Xiamen University (the 175th Hospital of Chinese PLA), NO.269, Zhanghua Middle Road, Zhangzhou, Fujian 363000 China

**Keywords:** Arsenic trioxide, Nilotinib, AMN107, CML-BC, Proliferation, Differentiation

## Abstract

**Aim:**

To determine the effects of arsenic trioxide (ATO) and nilotinib (AMN107, Tasigna) alone or in combination on the proliferation and differentiation of primary leukemic cells from patients with chronic myeloid leukemia in the blast crisis phase (CML-BC).

**Methods:**

Cells were isolated from the bone marrow of CML-BC patients and were treated with 1 μM ATO and 5 nM nilotinib, either alone or in combination. Cell proliferation was evaluated using a MTT assay. Cell morphology and the content of hemoglobin were examined with Wright-Giemsa staining and benzidine staining, respectively. The expression of cell surface markers was determined using flow cytometric analysis. The levels of mRNA and protein were analyzed using RT-PCR and Western blotting, respectively.

**Results:**

ATO and nilotinib alone or in combination suppressed cell proliferation in a dose- and time-dependent pattern (P < 0.01 vs. control). Drug treatments promoted erythroid differentiation of CML-BC cells, with a decreased nuclei/cytoplasm ratio but increased hemoglobin content and glycophorin A (GPA) expression (P < 0.01 compared with control). In addition, macrophage and granulocyte lineage differentiation was also induced after drug treatment. The mRNA and protein levels of basic helix-loop-helix (bHLH) transcription factor T-cell acute lymphocytic leukemia protein 1 (TAL1) and B cell translocation gene 1 (BTG1) were both upregulated after 3 days of ATO and Nilotinib treatment.

**Conclusions:**

Our findings indicated that ATO and nilotinib treatment alone or in combination greatly suppressed cell proliferation but promoted the differentiation of CML-BC cells towards multiple-lineages. Nilotinib alone preferentially induced erythroid differentiation while combined treatment with ATO preferentially induced macrophage and granulocyte lineage differentiation.

## Introduction

Chronic myelogenous leukemia (CML) consists of three distinct clinical stages, the chronic phase (CP), accelerated phase (AP) and blast crisis (BC) phase. The BC phase is the most aggressive and terminal phase [1]. At present, CML treatments, especially for patients in the BC phase, are limited. The pathological mechanism of CML in BC (CML-BC) is unclear; however, cytogenetic instability, DNA damage, impaired DNA repair, inhibited cell apoptosis, dysfunctional cell differentiation and drug resistance have been implicated in this disease [1,2]. Importantly, enhanced proliferative potential as well as differentiation arrest are recognized as the predominant phenotypes of CML-BC [3]. Inducing CML-BC cell differentiation in drug therapy may offer a promising approach for the treatment of CML patients in BC.

Previous studies have shown that the specific tyrosine kinase inhibitor imatinib (STI571) [4] and nilotinib (AMN107, trade name Tasigna) [5] are approved for the treatment of CML-AP or CML-BC patients. The second-generation tyrosine kinase inhibitor nilotinib appears to be effective and well-tolerated for those who are resistant to imatinib treatment [6,7]. However, BCR-ABL kinase domain mutations, which can occur before and during nilotinib therapy, have been indicated to affect the clinical efficacy of nilotinib [8]. Hence, combined drug applications may help overcome the drug resistance of nilotinib therapy alone. Recently, the anti-cancer agent arsenic trioxide (ATO) has been used for the treatment of patients with acute promyelocytic leukemia (APL) [9]. ATO yields complete remission in APL patients possibly through induction of cell apoptosis and differentiation [10]. In addition, a recent report demonstrated that combined treatment of interferon with ATO prolonged the survival of rodents with primary CML [11]. However, the potential effects of ATO and nilotinib, either alone or in combination, on CML-BC remains unclear.

In this study, we investigated the effects of ATO and nilotinib on the proliferation and differentiation of CML-BC cells isolated from CML patients in the BC phase. Our findings may provide basic evidence for understanding the molecular mechanisms of ATO and nilotinib therapy for CML patients in the BC phase.

## Results

### Effects of ATO and nilotinib on cell proliferation

**Figure 1:**
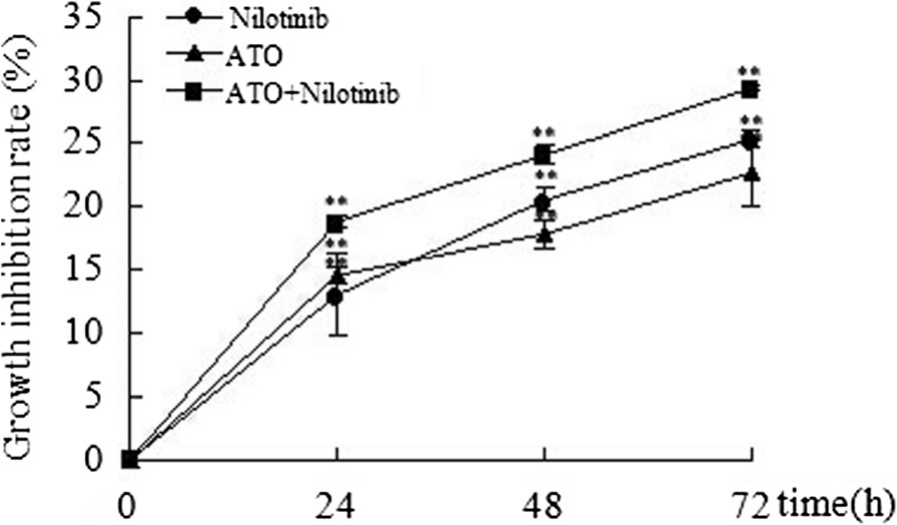
**Effects of ATO and nilotinib on cell proliferation.** Cells were treated with 1 μM ATO and 5 nM nilotinib alone or in combination for 24, 48 or 72 h, respectively. Cell proliferation was determined using a MTT assay. CML-BC cells were derived from five patients with CML-BC and data were calculated from five independent experiments. **P < 0.01 vs. control.

We first analyzed the effects of ATO and nilotinib alone or in combination on the proliferation of isolated CML-BC cells. The results demonstrated that ATO and nilotinib alone or in combination suppressed cell proliferation in a dose- and time-dependent pattern (P < 0.01 compared with control) (Figure 1). Compared with an single drug treatment, treatment with ATO plus nilotinib significantly reduced cell proliferation compared to the other groups (Figure 1) (P < 0.01).

### Morphological examination

**Figure 2:**
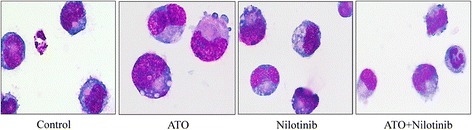
**Morphological examination of the effects of ATO and nilotinib on cell differentiation.** Cells were treated with 1 μM ATO and 5 nM nilotinib alone or in combination for 72 h. The cells were stained with Wright-Giemsa, and cell morphology was examined under light microscope at 1000x magnification.

Using Wright-Giemsa staining, we examined the morphology of cells following ATO and nilotinib treatment. As shown in Figure 2, control cells were relatively larger in size and the nuclei/cytoplasm ratio was also higher than treated cells. Untreated control cells showed round or oval shapes, chromatin was loosely and non-uniformly distributed in nuclei, the nucleus was large and clear, and cytoplasm was stained with blue. Following ATO or nilotinib treatment, cell size was greatly decreased. In addition, chromatin was condensed and the nuclei/cytoplasm ratio was reduced, suggesting that these cells were at an early or middle differentiation stage. In the ATO and nilotinib combination treatment group, cells showed early erythroid differentiation morphology, and a small proportion of cells underwent granulocyte differentiation.

### Effects of ATO and nilotinib on the hemoglobin content

**Figure 3:**
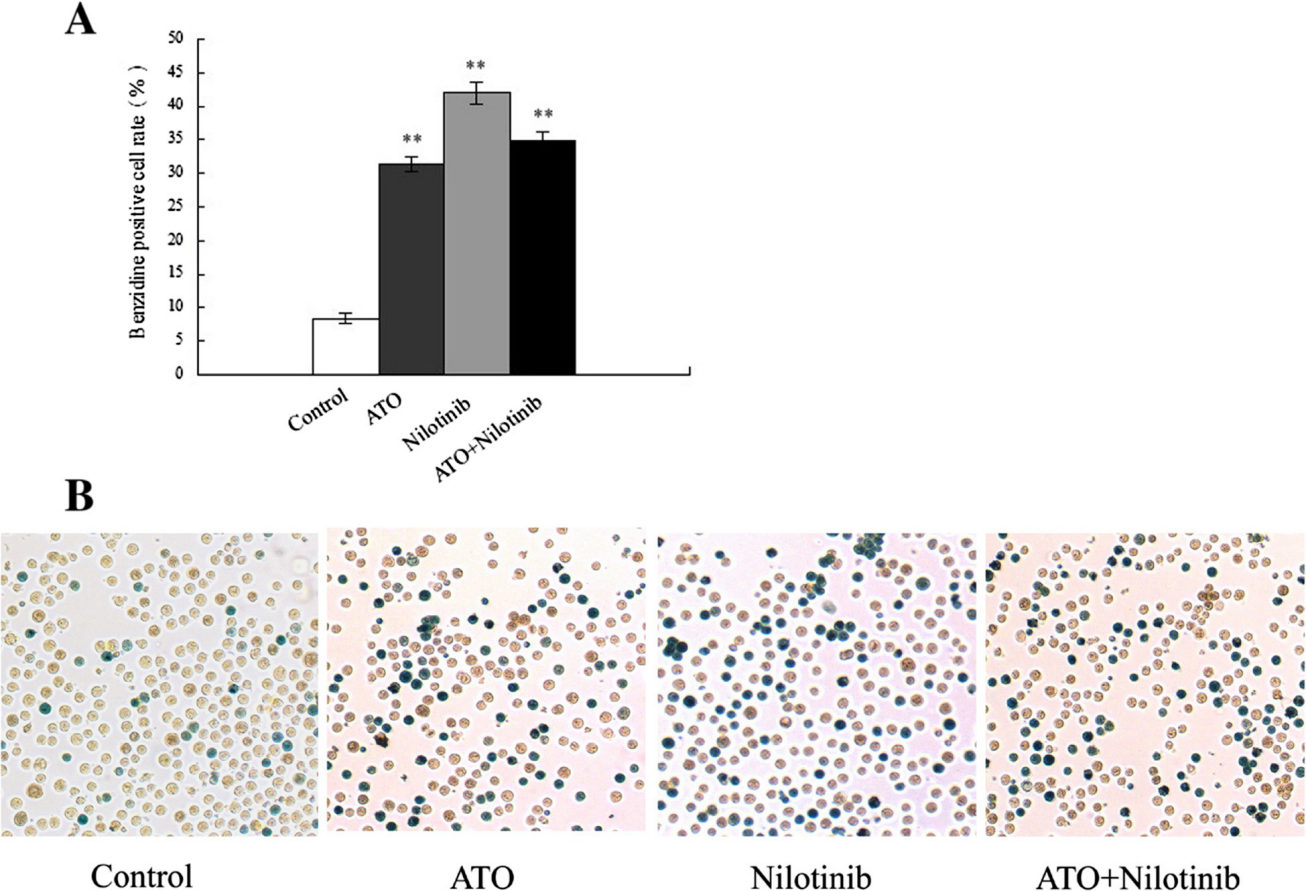
**Effects of ATO and nilotinib on hemoglobin content.** Cells were treated with 1 μM ATO and 5 nM nilotinib alone or in combination for 72 h. Then, cell samples were stained with benzidine. **(A)** The percentage of benzidine-positive cells was calculated. **P < 0.01 vs. control. **(B)** Representative data of benzidine staining (x200 magnification).

The cell hemoglobin content following ATO or nilotinib treatment was evaluated using benzidine staining. ATO or nilotinib treatment alone dramatically increased the percentage of benzidine-positive cells, and nilotinib appeared to be more efficient in upregulating the number of benzidine-positive cells (control, 8.41% ± 0.75%; ATO, 31.43% ± 1.15%; Nilotinib, 42.05% ± 1.69%; P < 0.01 vs. control) (Figure 3). Although combination treatment with ATO and nilotinib elevated the percentage of benzidine-positive cells, the effect was less than nilotinib treatment alone (ATO + nilotinib, 34.96% ± 1.12%). These results indicated that ATO and nilotinib treatment could increase CML-BC hemoglobin content.

### Effects of ATO and Nilotinib on the cell surface antigen expression

**Figure 4:**
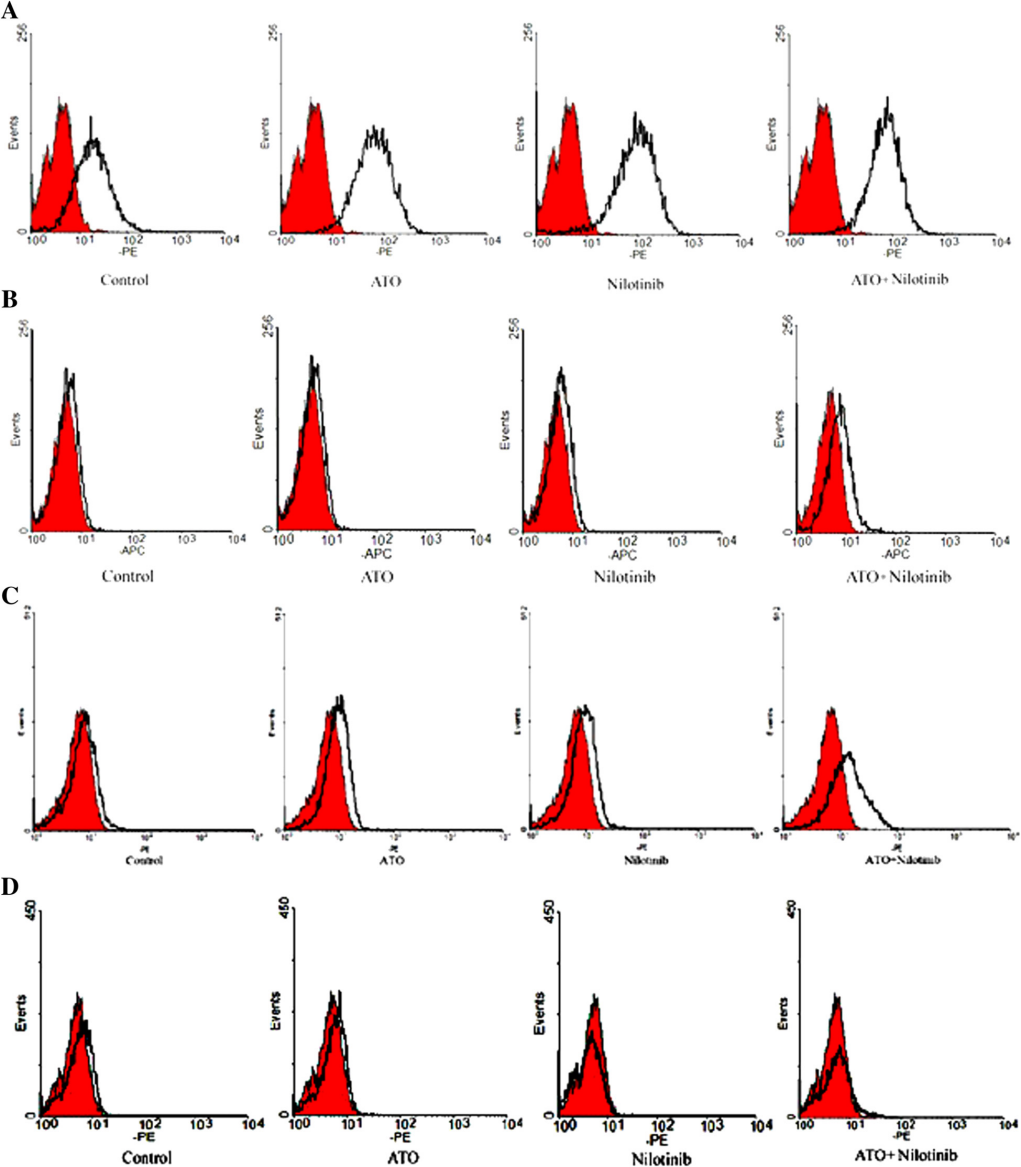
**Effects of ATO and nilotinib on cell surface antigen expression. A**: The level of GPA induced by ATO and nilotinib alone or in combination (P < 0.01 vs. control). **B**/**C**: ATO and nilotinib alone or in combination elevated the expression of CD41 and CD11b (P < 0.05). Combined treatment was more potent in upregulating CD41 and CD11b levels as compared with single drug treatment. **D**: the expression of CD14 after drug treatment as compared with control (P > 0.05).

We next determined the cell surface antigen expression, including GPA, CD41, CD11b and CD14, using flow cytometric analysis. As shown in Figure 4, a 72-h incubation with ATO and nilotinib alone or in combination significantly induced the level of GPA, erythroid lineage marker. Nilotinib had the greatest effect on GPA expression (Control, 17.85% ± 0.74%; ATO, 73.36% ± 1.28%; Nilotinib, 86.34% ± 0.52%; ATO + Nilotinib, 85.39% ± 1.32%; P < 0.01 vs. control). There was no significant difference in GPA expression between nilotinib treatment and ATO plus nilotinib treatment (P > 0.05). Similarly, ATO and nilotinib alone or in combination elevated the expression of CD41 and CD11b, which are macrophage and granulocyte lineage markers (P < 0.05). Combined treatment was more potent in upregulating CD41 and CD11b levels as compared with single drug treatment. There was no significant difference in the expression of monocyte biomarker CD14 after drug treatment as compared with control (P > 0.05). These findings suggested that ATO and nilotinib treatment could promote the erythroid lineage differentiation of CML-BC cells.

### Effects of ATO and nilotinib on TAL1 and BTG1 expressions

**Figure 5:**
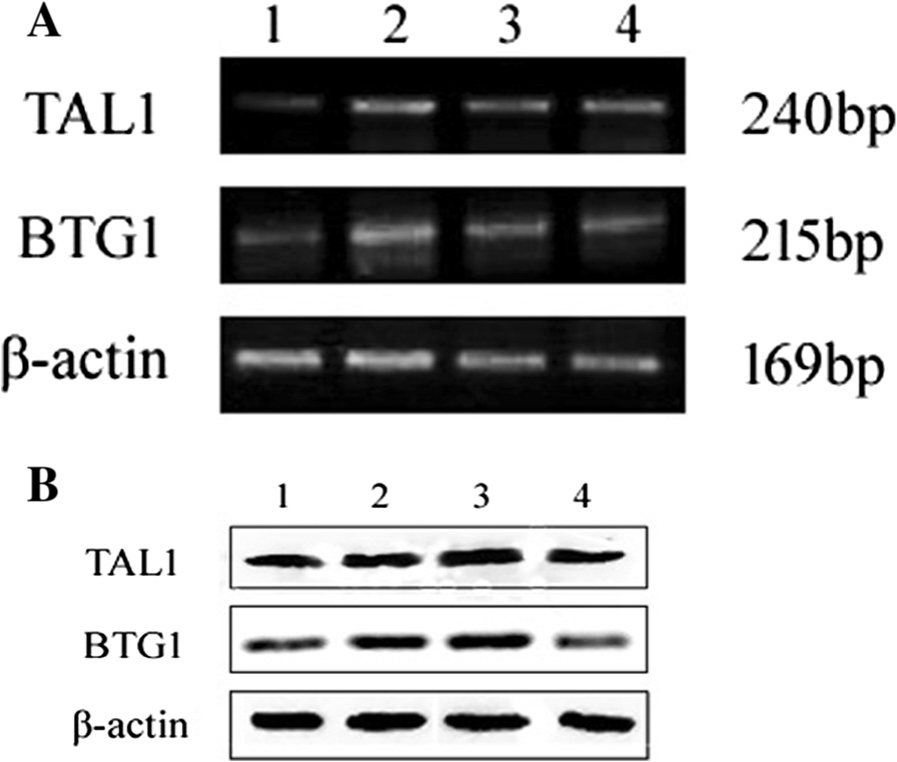
**Effects of ATO and nilotinib on the expressions of TAL1 and BTG1.** Cells were treated with 1 μM ATO and 5 nM Nilotinib alone or in combination for 72 h. The mRNA **(A)** and protein **(B)** expressions of TAL1 and BTG1 were evaluated by RT-PCR and Western blotting, respectively. β-actin was used as an internal control, and representative data were presented. **(A)** 1, Control; 2, Nilotinib treatment alone; 3, ATO treatment alone; 4, Nilotinib + ATO. **(B)** 1, Control; 2, ATO treatment alone; 3, Nilotinib treatment alone; 4, Nilotinib + ATO.

Finally, we examined the mRNA and protein expressions of TAL1 and BTG1 in cells following different treatments. The mRNA and protein levels of TAL1 and BTG1 were both upregulated after three days of ATO and nilotinib treatment, either alone or in combination (Figure 5). However, compared with the single drug treatments, combined treatment slightly decreased the expressions of TAL1 and BTG1. These results imply that TAL1 and BTG1 may be involved in ATO and nilotinib-mediated erythroid lineage differentiation.

## Discussion

Although the well-known tyrosine kinase inhibitor imatinib is effective for all three phases of CML, including CP, AP and BC, the response during advanced stages of CML are transient [12-15]. Nilotinib is a novel orally bioavailable inhibitor of the BCR-ABL tyrosine kinase and has a potency of approximately 20 times that of imatinib [16]. At present, nilotinib is used for imatinib-refractory CML [17]. However, the response to nilotinib therapy alone is limited, and thus combined drug therapy may be needed to avoid resistance. In the present study, we determined the influences of nilotinib and ATO, either alone or in combination, in cultured cells isolated from CML-BC patients.

CML-BC involves the accumulation of primitive hematopoietic cells arrested at an early stage of differentiation. In the patients in this study, a great proportion of cells in were in the early differentiation stage. Administration of ATO and nilotinib, alone or in combination, significantly suppressed cell proliferation but promoted erythroid differentiation and granulocyte differentiation. These findings are consistent with our previous study, which showed that nilotinib induced erythroid differentiation [18]. ATO is an anti-leukemic agent that leads to growth suppression and apoptosis induction CML K562 and human leukemia HL60 cells [19-21]. Our preliminary experiments demonstrated that a low dose of ATO (0.5-1 μM) induced differentiation of CML-BC cells, while ATO at a concentration over 1 μM dramatically inhibited cell growth and promoted cell apoptosis. In accordance with our findings, Miller et al. reported that the effects of ATO on K562 cells was dose dependent [22]. These findings provide further evidence of the efficiency of ATO and nilotinib in suppressing proliferation and promoting erythroid differentiation of CML-BC cells.

In order to understand the underlying mechanism of ATO and nilotinib-induced erythroid differentiation of CML-BC cells, TAL1 and BTG1 expressions were determined. *TAL1* gene, also known as *SCL* or *TCL5*, expression has been identified during the differentiation of erythroid, megakaryocytic, and basophilic lineages [23]. The production of erythroid and myeloid cells is dramatically decreased in *TAL1* silenced human hematopoietic cells [24]. In K562 cells, *TAL1* knockdown suppressed erythroid differentiation [25]. In addition, Aplan et al. reported that overexpression of TAL1 in K562 cells in creased the rate of spontaneous (i.e. in the absence of an inducer) erythroid differentiation [26]. In this study, ATO and nilotinib treatment promoted the erythroid differentiation of CML-BC cells and accompanied increased TAL1 expression. These evidences suggest that TAL1 may be a positive regulator of erythroid differentiation.

BTG1 serves as a Forkhead box, class O 3a (FoxO3a) target gene in erythroid differentiation [27]. Increased BTG1 expression has been observed in erythroid progenitors during erythroid differentiation [27]. In our previous study, we showed that FoxO3a activation might promote erythroid differentiation of CML-BC cells via down-regulating TAL1 expression [18]. In this study, increased BGT1 and TAL1 levels were detected in CML-BC cells following 72 h of nilotinib treatment. This discrepancy might be due to the prolonged nilotinib incubation (5 d [18] vs. 3 d) and/or increased drug dose (50 nM [18] vs. 5 nM) in our previous study. It is possible that TAL1 expression is upregulated during early erythroid differentiation, but downregulated during late stages of differentiation. Besides, the efficiency of ATO in increasing TAL1 and BTG1 expression appears to be less potent than that of nilotinib.

Here, we observed a synergistic effect of ATO and nilotinib treatment in suppressing CML-BC cell proliferation. Although ATO and nilotinib, alone or in combination, could induce the differentiation of CML-BC cells into multiple lineages, including erythroid, macrophage and granulocyte lineages, erythroid differentiation seemed to predominate. Interestingly, ATO and nilotinib did not have a synergistic effect in inducing erythroid differentiation. However, combined therapy showed increased efficacy in promoting macrophage and granulocyte lineage differentiation.

Collectively, our present study demonstrated that ATO and nilotinib, alone or in combination, suppressed proliferation and promoted differentiation, especially erythroid differentiation, of CML-BC cells. Our data may provide basic evidence for the clinical chemotherapy of CML patients in BC.

## Materials & methods

### Reagents

ATO was purchased from Beijing SL Pharmaceutical Co., Ltd in Beijing, China. RPMI-1640 culture medium and fetal bovine serum (FBS) were obtained from GIBCO, Life Technologies (Carlsbad, CA, USA). The First Strand cDNA Synthesis Kit and mouse anti-human monoclonal primary antibodies against CD41, GPA and CD11b were bought from Biolegend (San Diego, CA, USA). Mouse anti-human monoclonal primary antibodies against TAL1 and BTG1 were purchased from Santa Cruz Biotechnology (Dallas, Texas, USA). All of the other reagents were obtained from Sigma-Aldrich (St. Louis, MO, USA) unless stated.

### Cell culture

CML-BC cells were derived from five patients with CML-BC in the No. 175 PLA Hospital of China. CML-BC was diagnosed based on the bone marrow smear and philadelphia chromosome analysis. Bone marrow mononuclear cells were isolated by density centrifugation (20 min at 500 g) using lymphocyte separation medium. The middle layer mononuclear cell samples were washed three times with phosphate buffer solution (PBS) and resuspended with culture medium containing 10% FBS and 1% antibiotics. The single-cell suspension was adjusted to an appropriate density and seeded onto 96-well plates at a density of 5–6 cells/well. After 7–10 days of *in vitro* culture, the well with single clone formation was sub-cloned. This procedure was repeated for three to five times until the positive rate reached 100%.

### Experimental assignment

Cells were randomly divided into four groups with three wells in each group. Control cells were maintained with RPMI 1640 culture medium. In the ATO and nilotinib groups, cells were treated with 1 μM ATO and 5 nM nilotinib, respectively. In the ATO + nilotinib group, cells were incubated with 1 μM ATO plus 5 nM nilotinib.

### Evaluation of cell proliferation

To evaluate cell proliferation, a 3-(4,5-dimethylthiazol-2-yl)-2,5-diphenyltetrazolium bromide (MTT) assay was used. Cells were seeded onto a 96-well plate at a density of 3 × 10^3^ cells/well. After seeding, cells were treated with 200 μL of culture medium containing ATO, nilotinib or ATO plus nilotinib. Control cells were incubated with the same volume of culture medium. After 24-, 48-, or 72-h of incubation at 37°C in a 5% CO_2_ incubator, 20 μL of MTT (5 mg/mL) was added to each well. After a subsequent 4 h of incubation, the medium was removed and 150 μL of dimethyl sulfoxide (DMSO) was added to each well to resuspend the MTT metabolic product. The absorbance of the dissolved formazan was measured at 570 nm using a scanning microplate spectrophotometer.

### Wright-Giemsa staining

Cells were collected by centrifugation, and then cell smears were prepared. After fixation in methanol-glacial acetic acid solution for 10 min, cells were stained with Wright-Giemsa for 1 min and then washed with PBS. Cell samples were dried in air, and the cell morphology was examined under light microscope.

### Benzidine staining

A total of 0.5 mL of cell suspension was mixed with 14 μL of benzidine dye and 0.15 μL of 30% hydrogen peroxide. After 5 min, 0.15 μL of 5% nitroso iron sodium hydride was added into the mixture. After a 10–15 min incubation, cells were examined under a phase contrast microscope (Motic AE30). The blue-brown cells containing hemoglobin were identified as positive cells, and other cells were recognized as negative cells. A total of 300 cells were counted, and the percentage of positive cells was calculated from three independent experiments.

### Cell surface antigen

The expression of cell surface antigen was evaluated by flow cytometric analysis. Briefly, after three days of drug treatment, cells were collected by centrifuging at 1,500 rpm for 5 min. The supernatant was discarded and cells were washed twice with cold PBS. The cell number was adjusted to 0.1-1 × 10^6^ cells. For each experimental group, cell samples were divided into two tubes containing 50 μL of cell sample each. One tube was used to evaluate cell surface antigen and the other was used as an isotype control. To assess the expression of specific cell surface antigen, cells were incubated with primary antibody against target antigen at 4°C for 60 min. In the isotype control group, cells were incubated with a fluorescein-labeled isotype control antibody under the same incubation conditions. Samples were acquired with a Partec CyFlow Space flow cytometer (Partec GmbH, Germany), and data were analyzed using WinMDI version 2.9 software.

### Reverse transcription polymerase chain reaction (RT-PCR) analysis

After three days of drug treatment, total RNA was extracted from cells using Trizol reagent. cDNA was synthesized according to the instructions of the First Strand cDNA Synthesis Kit. PCR amplification was performed on the BTG1 gene upstream primer (5′-CTG CAG ACC TTC AGC CAG AG-3′) and downstream primer (5′-GGG TCA ACC CAG AGT GTG AG-3′), TAL1 gene upstream primer (5′-TTC GTG AGC CCC ATC TTC AC-3′) and downstream primer (5′-ACA GCC ACA GGC TTA GGA AG-3′), and β-actin gene upstream primer (5′-CAC ACA GGG GAG GTG ATA GC-3′) and downstream primer (5′-GAC CAA AAG CCT TCA TAC ATC TCA-3′). The PCR reaction included 10 μL of PCR Master mix, 1 μL of each of the upstream and downstream primers, 4 μL of template cDNA, and 6 μL of ddH_2_O in a total volume of 20 μL. The PCR reactions started with an initial denaturation at 94°C for 2 min, followed by 28 cycles of 94°C for 30 s, 55°C for 30 s, and an elongation step of 70°C for 2 min. After gel electrophoresis in 1.5% agarose, ethidium bromide-stained bands were visualized with ultraviolet transillumination.

### Western blotting

Total protein was extracted from cells following drug treatment for three days using lysis buffer supplemented with phenylmethylsulfonyl fluoride (PMSF). After centrifuging at 12,000 rpm for 5 min, the supernatant was collected and the protein concentration was measured using a Bradford assay. Equal amount of protein extract was separated by sodium dodecyl sulfate-polyacrylamide gel electrophoresis (SDS-PAGE), transferred to a PVDF membrane, blocked for 1 h, and then incubated with primary antibodies at room temperature for 1 h. The primary antibodies, including anti-BTG1 (1: 200 dilution), anti-TAL1 (1: 200 dilution) and anti-β-actin (1: 4000 dilution) antibodies, were diluted in Tris buffered saline plus Tween-20 (TBS-T). After washing with TBS-T, membranes were incubated with a secondary antibody for 1 h. Immunobands were visualized using an ECL kit (Xiamen Lulong Biotech Co., Ltd., Xiamen, China). The housekeeping protein β-actin was used as an internal loading control.

### Statistical analysis

Data were analyzed by SPSS17.0 software and presented as means ± standard deviation (SD). Statistical significance between control and sample groups was determined using Student *t*-tests. *P* < 0.05 or 0.01 was recognized as statistically significant.
